# O universo masculino no domicílio: a visão dos homens acerca do Programa Melhor em Casa

**DOI:** 10.26633/RPSP.2019.320

**Published:** 2019-10-18

A *Revista Panamericana de Salud Pública* informa aos seus leitores sobre erro no seguinte artigo indicado pelos autores, em relação ao sobrenome de alguns deles:

Araújo JF, Freitas FBD, Cavalcante JRD, Freitas JMS, Silva AE, Santos WP. O universo masculino no domicílio: a visão dos homens acerca do Programa Melhor em Casa. Rev Panam Salud Publica. 2018;42:e123. https://doi.org/10.26633/RPSP.2018.123

No artigo original publicado em dezembro de 2018 os sobrenomes dos autores aparecem como:

Jocelly Ferreira de Araújo, Fernanda Beatriz Dantas de Freitas, Joseane da Rocha Dantas Cavalcante, Jucicleia Maiara da Silva Freitas, Alexandre Ernesto da Silva e Wallison Pereira dos Santos

Houve um erro no sobrenome dos seguintes autores:

Jocelly de Araújo Ferreira, Alexandre Ernesto Silva, Joseane da Rocha Dantas Cavalcanti.

A forma correta de citar o artigo é:

Ferreira JA, Freitas FBD, Cavalcanti JRD, Freitas JMS, Silva AE, Santos WP. O universo masculino no domicílio: a visão dos homens acerca do Programa Melhor em Casa. Rev Panam Salud Publica. 2018;42:e123. https://doi.org/10.26633/RPSP.2018.123

